# Cellular-Based Immunotherapies for Patients with Glioblastoma Multiforme

**DOI:** 10.1155/2012/764213

**Published:** 2012-02-28

**Authors:** Xun Xu, Florian Stockhammer, Michael Schmitt

**Affiliations:** ^1^Center for Biomaterial Development and Berlin-Brandenburg Center for Regenerative Therapies, Institute of Polymer Research, Helmholtz-Zentrum Geesthacht, 14513 Teltow, Germany; ^2^Department of Neurosurgery, University of Göttingen, 37073 Göttingen, Germany; ^3^Department of Internal Medicine V, Hematology, Oncology and Rheumatology, University of Heidelberg, 69120 Heidelberg, Germany

## Abstract

Treatment of patients with glioblastoma multiforme (GBM) remains to be a challenge with a median survival of 14.6 months following diagnosis. Standard treatment options include surgery, radiation therapy, and systemic chemotherapy with temozolomide. Despite the fact that the brain constitutes an immunoprivileged site, recent observations after immunotherapies with lysate from autologous tumor cells pulsed on dendritic cells (DCs), peptides, protein, messenger RNA, and cytokines suggest an immunological and even clinical response from immunotherapies. Given this plethora of immunomodulatory therapies, this paper gives a structure overview of the state-of-the art in the field. Particular emphasis was also put on immunogenic antigens as potential targets for a more specific stimulation of the immune system against GBM.

## 1. Introduction

The primary brain tumor, glioblastoma multiforme (GBM), occurs in 3 to 4 adult patients per 100,000 inhabitants in Europe, thus being the most common and life threatening primary brain tumor [1]. GBM is invasive and infiltrates the surrounding brain tissue.

GBM is most common in adults older than 50 years and affects more men than women. Furthermore, around 9% of childhood brain tumors are GBMs. The median survival from the time of diagnosis without any treatment is 3 months. The major prognostic factors are age and Karnofsky performance status (KPS) at the time of diagnosis [2].

## 2. Standard Treatment of Glioblastoma (GBM)

### 2.1. Primary Treatment

Although GBM has a typical appearance in MRI, histological diagnosis is mandatory for proper diagnosis. A treatment option is gross tumor resection (GTR), which involves the contrast enhancing tumor in to the MRI without causing additional neurological deficit [3]. The extent of resection can be optimized using fluorescence-guided resection [4], which probably includes noncontrast enhancing tumor part and can be visualized by aminoacid positron emission tomography [5]. Placing carmustine-loaded wafers in the resection cavity has shown to prolong survival rates [6]. However, the clinical benefit seems to be limited to patients with GTR and good KPS [7]. If GTR cannot be achieved and the tumor mass does not cause a midline shift, stereotactic serial biopsy is a safe procedure to enable histological and genetic diagnosis [8–10].

Following surgery, a typical treatment consists of concomitant temozolomide and 60 Gy radiotherapy of the tumor region for six weeks followed by 6 adjuvant cycles of temozolomide [11, 12]. After applying this treatment, tumors with methylated promotor for O(6)-methyl-guanine DNA methyltransferase (MGMT) appear to have a favorable clinical course with a median overall survival of 21.7 months [13].

Although long-term adjuvant temozolomide is safe [14], there is no evidence that the continuation of temozolomide beyond six cycles gives any additional benefit.

### 2.2. Recurrent GBM

In recurrent GBM, surgery is an optional treatment. However, there is no evidence for the clinical benefit of second surgery. According to retrospective studies, a second resection should be restricted to patients with good KPS and feasible systemic salvage treatment [15, 16]. Alternatively, conformal reirradiation may be administrable [17, 18].

For systemic treatment of recurrent glioblastoma, bevacizumab is FDA approved but only with class 2 evidence [19–22]. In Europe, bevacizumab remains off-label treatment for glioblastoma patients. Rechallenge with TMZ is an alternative to bevacizumab. Thereby, protocols vary from dose-intensified treatment to metronomic schedules [23–26]. However, there are no positive randomized controlled trials defining a standard treatment in recurrent glioblastoma. However, salvage treatment should be applied as long as the patient's condition has not declined [27].

Due to the limited treatment options for GBM patients, there is a fervent need for novel therapies such as immunotherapies. However, the brain is known as one of the immunologically privileged sites and is able to tolerate the introduction of antigen without eliciting an inflammatory immune response [28]. Thus, immunotherapy for brain tumor seems to constitute a “mission impossible.” Fortunately, it is now known that the central nervous system at least maintains a reciprocal communication network with the immune system. Infectious or experimental autoimmune encephalomyelitis animal models allow us to understand better how the immune system operates in the brain [29]. Therefore, immunotherapy offers the opportunity to allow the patient's immune system a chance to eliminate the tumor. The strength of immunotherapy with DCs has been demonstrated on the Food and Drug Administration (FDA) approval of DCs as “Provenge” for prostate cancer [30]. As for GBM, it has been demonstrated that it efficiently treats relatively small tumors in experimental animal models.

This paper focuses on the cellular-based immunotherapy for brain cancers with emphasis on GBM. We will also highlight some of the possible directions that may be taken in the immediate future to improve this therapeutic option.

## 3. Immunotherapy

There are two important basic strategies for immunotherapy. Firstly, adoptive immunotherapy, which means the passive administration of sensitized immune cells to patients. Secondly, the strategy of “active immunotherapy” is based on the boosting of antitumor T-cell responses by antigen-presenting cells (APCs), especially by dendritic cells (DCs).

### 3.1. Adoptive Immunotherapy

In adoptive immunotherapy, *in vitro* activated immune cells are administered to tumor-bearing patients. Lymphokine-activated killer (LAK) cells, which were generally obtained by cultivating peripheral lymphocytes in the presence of T-cell growth factor interleukin-2 (IL-2) and other cytokines. These LAKs showed cytolytic properties but not specifically against tumor cells [31–42]. A novel therapeutic option was to collect lymphocytes from lymph nodes or peripheral blood mononuclear cells (PBMCs) after peripheral injection of irradiated autologous tumor cells and granulocyte-macrophage colony-stimulating factor (GM-CSF), stimulating them *in vitro*, and subsequently reinjecting them [43–47]. Allogeneic cytotoxic T lymphocytes (CTLs) stimulated by the patient's autologous lymphocytes have been tested for recurrent GBM as well [48]. They were generated by *ex vivo* antigenic stimulation of PBMCs. As Quattrocchi et al. have shown in a pilot study, CTLs can also be amplificated from tumor-infiltrating lymphocytes (TILs) in the presence of IL-2 [49].

Injection of CTLs or TILs appeared to allow higher objective responses compared to LAKs in some GBM patients. Due to the large variability observed between patients and the limited number of patients, the correlation between the number of effector cells, their cytotoxic activity, and the clinical outcome is still not clear. Kronik et al. have predicted that GBM would be eradicated by new dose-intensive strategies, for instance, 3 × 10^8^ CTLs every 4 days for small tumor burden or 2 × 10^9^ CTLs infused every 5 days for larger tumor burden [50]. Interestingly, in several clinical trials with LAK and CTL therapy, the eosinophilic infiltration at the tumor site and in cerebrospinal fluid (CSF) could be observed [38, 40, 48, 49]. The impact of chemotherapy or corticosteroids on the efficacy of the treatment might also be questioned. These drugs were completely avoided in some trials according to their immunosuppressive properties [31, 39, 41, 42]; however, other studies have shown no influence of steroids or chemotherapy on the stimulation and the cytotoxic activity of the effector cells [32, 34, 35].

Progress in the treatment of brain tumors using immunotherapy is slowly moving forward. Initial attempts used nonspecific approaches, like adjuvants and, LAK cells were only minimally effective. Nowadays, the general focus is directed towards specific cellular approaches including TILs and CTLs, and alloreactive CTL stimulated by mixed lymphocyte reactions. All of these approaches have yielded some clinical success. GBM cells seem to have a plethora of tumor-associated antigens. Active immunization with autologous DCs that have been loaded with tumorantigens also appear to generate long-term survivors. Identification of other strategies that can be combined with immunotherapy approaches might improve our success against GBM.

### 3.2. Active Immunotherapy

#### 3.2.1. Active Immunotherapy in GBM Using Autologous Tumor Cells (ATCs)

Autologous tumor cells are removed from patients during surgery. Whole cells, parts of cells, or antigens can be used to create a vaccine to a specific tumor. To date, there are at least eight high qualified trials, which focused on the GBM treatment were reported [51–58]. One phase I clinical trial [58], two cases report [51, 54], and five pilot studies of antitumor vaccination [52, 53, 55–57] were included, and the vaccination was repeated in all of these studies. ATCs are generally inactivated by radiation, sometimes genetically modified [51, 54, 56], and could be infected with a virus [52, 55] to enhance the immune response. The strategy of using antisense oligonucleotides for insulin growth factor receptor 1 for ATCs prior to implantation was applied in one pilot study [53]. The cells were injected either subcutaneously or intradermally. In three studies, injections of ATCs were given concomitantly with IL-2 [51], IL-4 [54] or B7-2, and GM-CSF infusions [56]. Different amounts of cells were given for vaccination.

At least half of the patients in these studies showed an induction of immune responses both in peripheral blood and tumor site [53, 55]. Toxicity was addressed in all trials without any severe adverse events*.* Beside an immune response, a clinical response was demonstrated to be associated with survival benefit in five studies with three complete responses (CRs), four partial responses (PRs), two minor responses (MRs), and six stable diseases (SDs) in a total of 48 GBM patients [53–55, 57, 58].

#### 3.2.2. Active Immunotherapy Using Dendritic Cells

**Figure 1:**
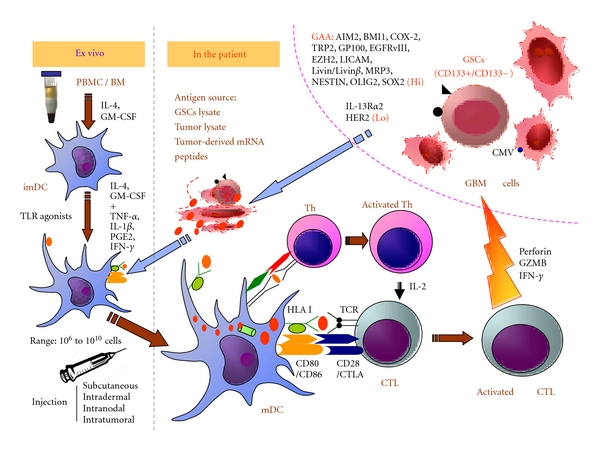
DC-based active immunotherapy for GBM. DCs display a unique capacity to induce and to maintain T-cell responses. Mature DCs are generated from PBMC *in vitro* in the presence of IL-4, GM-CSF, TNF-alpha, IL-1beta, PGE2, IFN-gamma, and other cytokines, in addition to TLR agonists. Subsequently, they are loaded with GBM or glioblastoma stem cell lysates, GBM-associated antigen-derived peptides, protein, or RNA. Due to their high surface expression of HLA-peptide-complexes and costimulatory molecules, DCs could efficiently activate and expand CD8^+^ CTLs and CD4^+^ Th cells. CD8^+^ CTLs are able to recognize and eliminate tumor cells, especially the GBM stem cells (CD133). CD4^+^ Th cells enhance the capacity of DCs to induce CTLs by the interaction between CD40 on DCs and CD40 ligand on activated CD4^+^ T cells. In addition, CD4^+^ T cells help in the maintenance and expansion of CTLs by secreting IL-2. CTLs: cytotoxic T cells; imDC: immature dendritic cells; GZMB: granzyme B; GSCs: glioblastoma stem cells, HLA: human leukocyte antigen; IL: interleukin; IFN: interferon; mDC: mature dendritic cells; PBMC: peripheral blood mononuclear cells; TCR: T-cell receptor; Th: T helper cell; TLR: toll-like receptor.

Dendritic cells (DCs) are professional antigen-presenting cells (APCs), which play a key role in eliciting, maintaining, and regulating T-cell responses [59, 60]. DCs are present in almost all organs, even in immune-privileged sites such as the central nervous system, testis, and ovaries. DCs can be generated not only from lymphoid organs but also from the blood or lymph. DC vaccines are attractive and now widely used in GBM active immunotherapy based on their various antitumor effects [Figure 1].

In Table 1, we summarized 15 clinical trials reported so far including 316 patients [61–75]: eight phase I trials [62, 64, 65, 69–72, 75], six phase I/II trials [61, 63, 66, 68, 73, 74], and one phase II trial [67]. Monocyte-derived DCs were used for most of the clinical trials. The preparation of DC is now well established, and a sufficient number of DC can be generated for injections into patients [76, 77]. Immature DCs were widely used in older trials [67, 69, 70, 74, 75]; however, some trials have used maturation factors like TNF-alpha [71], toll-like receptor (TLR) agonists: penicillin-killed streptococcus pyogenes (OK-432) [68] and imiquimod [62, 64], TLR ligand: poly ICLC [61, 62], IFN-gamma and TNF-alpha in combination with IL-4-secreting fibroblasts [78]. In several trials, DCs were matured using cocktails with IL-1beta, TNF-alpha, PGE2, or IFN-gamma [7, 63, 64, 74]. The number of DCs injected ranged from 1 × 10^6^ to 1 × 10^10^. The frequency of the injections was highly variable. One phase I study focused on the dose of DCs, which did not result in any dose-limiting toxicity [69]. The sources of antigen were quite different: autologous tumor lysates, apoptotic glioma cells peptides eluded from ATCs, synthetic peptides, defined peptides, mRNA derived from ATCs, and irradiated single-cell suspension of ATCs. For vaccinations using ATCs, the ATCs were fused [71] or incubated with DC. Defined peptides were derived from EGFRvIII, the particular target as its frequent expression in GBM [65, 79]. Vaccines were injected intradermally, intranodally, or subcutaneously. Moreover, in one phase I/II trial, some patients even received intratumoral injections [74].

From all of these clinical studies, only one patient had a large residual tumor and a perilesional edema suffered grade IV neurotoxicity (stupor) [68]. A peripheral immune response such as DTH (delayed-type hypersensitivity) lymphocyte infiltrations, particularly CD8^+^ cells, can be observed in more than half of patients. Activated CD8^+^ CTLs efficiently recognize and destroy tumor cells, which expose peptides derived from tumor-associated antigens (TAAs) in human leukocyte antigen (HLA) class I molecules [80]. CD4^+^ T cells recognizing peptides in the complex of HLA class II molecules also play an important role in antitumor immunity [81]. CD4^+^ T cells improve the capacity of DCs to induce CTLs by the interaction between CD40 on DCs and CD40 ligand on activated CD4^+^ T cells. In addition, CD4^+^ T cells help to maintain and expand CTLs by secreting cytokines such as IL-2. Beside their extraordinary capacity to elicit T-cell responses, DCs efficiently improve the immunomodulatory and cytotoxic potential of natural killer cells, which are also involved in the elimination of tumor [82, 83]. Furthermore, DCs can also directly mediate tumor-directed cytotoxicity [84].

Almost fifteen studies have reported on a survival benefit of patients receiving immunotherapies when compared with historical cohorts [61–70, 73–75]. Liau et al. [69] vaccinated four patients showing an increase of intratumoral infiltration by lymphocytes after vaccination at a time when the tumor was minimal. T-cell infiltration correlated with a decrease in intratumoral TGF-beta and was associated with a better survival. Patients without T-cell infiltration showed a reverse effect. Combined intravenous and intracranial administration of ATCs gave a superior response when compared to intravenous injection only [68]. Wheeler et al. reported on the large cohort of 34 GBM patients demonstrating that responders had an increase of IFN-gamma after vaccination when compared with the IFN-gamma level before vaccination using *in vitro* PBMC stimulation. Moreover, responders to vaccination showed a better response to chemotherapy which was delivered in a second phase [67]. Recent phase I and phase I/II studies with 10 newly diagnosed GBM and 13 recurrent GBM, vaccinated intranodally with autologous tumor lysate pulsed on DCs after radiation and chemotherapy or synthetic peptides for GAA epitopes showed a good immune response and a prolonged survival [61, 85].

As a consequence, active immunotherapy appears to have a beneficial effect in some patients, particularly in those with a limited tumor, without causing major toxicity. Both clinical trials using ATCs and DC demonstrate induced immune responses (DTH reaction, tumor infiltration, and/or anti-tumor responses of PBMC) and some clinical responses. The important take-home message for DC vaccination is that no dose-related toxicity has been demonstrated [69]. In addition, it seems better to use mature DC compared to immature DC. Due to the large variability of protocols tested, the source of ATCs, and the type and the cell number of DC injected, the type of adjuvants, no proven approach can be presented so far.

Various antigen sources can be used for DC active immunotherapy. Peptides are very popular; however, loading DCs with peptides requires a large culture of autologous tumor cells, which is a complex process. To break this limitation, some trials load DCs with tumor lysate instead of eluted peptides. Yu et al. [75] found T-cell-mediated cytotoxicity in 60% of the patients after immunization with tumor lysate-loaded DCs, a success rate higher than the 40% value seen with eluted peptides by the same team [70]. In another phase I/II trial, 24 patients with recurrent malignant gliomas were treated with intradermal or intratumoral (Ommaya reservoir) injections of DCs loaded with tumor lysate. Some patients also received intratumoral injections. One PR and three MR were observed [68]. A novel development in cancer vaccines consists of fusing tumor cells with DCs, a strategy that has been associated with clinical responses in patients with glioma [71].

#### 3.2.3. Antigens for GBM Immunotherapy

**(1) Glioblastoma-Associated Antigens (GAAs)**

Recently, many efforts have been made to identify tumor-associated proteins as targets of tumor-reactive T cells and to define peptide motifs within these proteins constituting T-cell epitopes. In this paper, we focus on glioblastoma-associated antigens (GAAs), which have already been used for DC-based vaccination trials enrolling GBM patients. GAAs such as EGFRvIII, EphA2, GP100, HER2, MAGE-1, IL-13R*α*2, SOX11, and TRP2 [86–89], which were frequently overexpressed in GBMs, were able to initiate immune responses. Other antigens associated with GBM have been described including survivin, WT1, SOX2, AIM2, SART1, SART2, and SART3 [90]. T cells directed against IL-13R*α*2 and EphA2 have been demonstrated in the PBMCs of a long surviving patient with anaplastic astrocytoma, showing that a spontaneous immune reaction can be observed in high-grade glioma [91]. Many glioblastoma-associated antigens were identified within either glioblastoma cell lines or GBM cells, such as ART and SART [92]. “Cancer-testis antigens” are differentially expressed in testis and tumors including MAGE-1, GAGE-1, and NY-ESO-1. These antigens were found in terminally differentiated melanocytes and also in GBMs [93–95]. TRP-1 and TRP-2 were not found in the testis but were detected in normal cells like melanocytes as well transformed tissues like melanomas and glioblastomas [89]. Since melanoma and glioblastoma cells share a common embryonic neuroectoderm precursor, it is not that surprising that these two cancer types share many common antigens. Here, we summarized the key GAAs in Table 2 [79, 89–123].

**(2) Viral Antigens**

**Figure 2:**
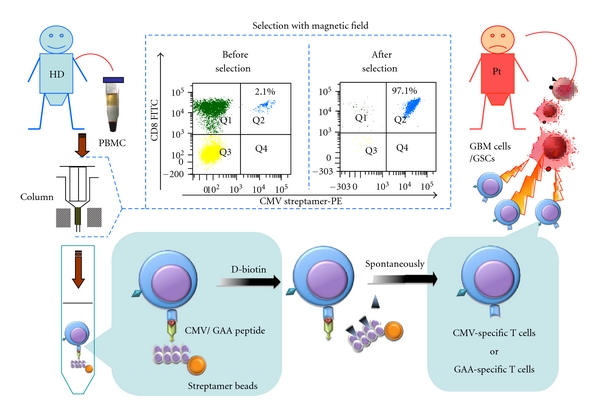
Adoptive immunotherapy for GBM patients with CMV or GAA peptides. CMV and GAAs are highly expressed in GBM, but neither in healthy brain tissue, nor in nonmalignant brain tumors. Therefore, GAAs constitute good targets for immunotherapy of GBM patients. The streptamer technology offers the advantage of selecting CMV- or GAA-specific CD8^+^ CTLs at the good manufacturing practice (GMP) level *in vitro*. PBMCs from healthy donors are collected and isolated by streptamer beads. Noninduced antigen-specific T cells are purified and accumulated through a magnetic field and released by D-biotin from the streptamer complex. Subsequently, these cells are administered to the GBM patient. CMV/GAA-specific cytotoxic T cells can recognize the target antigens which are presented on the surface of GBM cells or GSCs. Cytotoxicity is exerted directly through the Fas or perforin pathway and/or indirectly by the release of cytokines. CMV: cytomegalovirus; GAA: glioblastoma associated antigen; GBM: glioblastoma multiforme; GSCs: glioblastoma stem cells; HD: healthy donor; PBMC: peripheral blood mononuclear cells; Pt: patient.

Viral antigens act as good targets for anti-infectious immunity. Moreover, many viruses such as HTLV-1, hepatitis B and C virus, and EBV/JCV play a critical role in several human cancers as well. Cytomegalovirus (CMV, a common, typically harmless herpes virus) is frequently detected within chronically immunosuppressed patients. It is thought that up to 90% of the population might be chronically infected with CMV. However, our immune system keeps them under tight control. GBM patients are considered to be immunosuppressed through many mechanisms [124]. So the CMV can revive whenever the immune system is impaired. In 2002, Dr. Cobbs et al. [125] linked CMV with human GBM. They analyzed GBM samples from 22 patients and found that all of them harbored CMV. 80% people have this virus, which remains in the body for remaining lifespan. Whether CMV directly causes GBM is still a hot topic and is also controversial. The possibility that CMV attaches itself to GBM via the platelet-derived growth factor alpha allows some interesting therapies to be explored. One CMV antigen, pp65, induced a HLA-A2 restricted immune response in a GBM patient [126]. Freshly isolated GBM samples seem to highly express this CMVpp65 antigen, but cell lines lose this ability [127]. If a high number of GBM cells harbor CMV or express CMV antigen* in vivo*, this might open the door towards developing CMV peptides to vaccinate against the virus and the tumor at the same time [Figure 2]. Currently, after learning about Dr. Cobbs's work, Dr. Mitchell and his colleagues first confirmed the basic findings. They discovered CMV in the tumors of more than 90% of those patients with GBM, but not in healthy brain tissue nor in nonmalignant brain tumors [127]. Then used DC-based vaccines-targeting CMV antigens to treat 21 patients who had been diagnosed with GBM. Allogeneic CMV-specific CTLs have been used for treating glioma patients by the research group from Pennsylvania State University [128, 129]. Yao et al. [130] and Schmitt et al. [131] showed that the streptamer technology offered the advantage of selecting CMVpp65-specific CD8^+^ CTLs at the good manufacturing practice level *in vitro*. This strategy might then be used for adoptive immunotherapy for GBM patients in the future [Figure 2]. CMV-specific T cells might constitute a key of the useful immunological tool to attack GBM.

### 3.3. Role of Regulatory T Cells and Th17 Cells in Immunotherapy

CD4^+^ regulatory T cells (Tregs) play a key role in maintaining immune homeostasis. They have been well characterized as a distinct subpopulation of T cells due to the identification of the forkhead box transcription factor 3 (Foxp3) as an essential transcription factor in Tregs [132]. The investigation of Treg in brain tumor has blossomed in the last five years. The CD4^+^, CD25^+^ (IL-2R*α* ^+^), and Foxp3^+^ Tregs were most frequently found in GBM but very rarely in low-grade astrocytomas and were not present in normal brain tissue. Treg infiltration differed significantly in the brain tumor according to lineage, pathology, and grade. Under the microenvironmental conditions in the GBM patients, Tregs work in several ways to inhibit the effect of T-cell response and act as immune suppressors [133–135]. This might cause the failure of elimination of GBM with glioblastoma infiltrating lymphocytes in clinical trials. Options to eliminate Treg function will likely improve clinical results in future trials. Daclizumab is an approved antibody against IL-2R, which can be used for Treg elimination. Tregs share a common early-stage pathway with another type of CD4^+^ IL-17A^+^ T-helper cell, called Th17 cells [136, 137]. Naive T cells upon exposure to antigen and TGF-beta can generate mouse Th17 cells, but not human Th17 cells. For generating Th17 cells, the presence of IL-6 is required. Both cytokines are produced by GBMs. In a melanoma-bearing mouse model, Th17 cells could be used to clear large-established tumor cells [138]. To date, the presence of Th17 cells was confirmed in both human and mouse glioma as well [139], but their beneficial or inhibitory actions have not been fully understood.

### 3.4. Myeloid-Derived Suppressor Cells (MDSCs)

GBM patients are immunosuppressed and have more circulating myeloid-derived suppressor cells (MDSCs) when compared to normal donors. Interestingly, MDSCs might be generated from glioma-conditioned monocytes *in vitro* [140]. As Raychaudhuri et al. reported in 2011, GBM patients have increased MDSCs counts (CD33^+^ HLA-DR^−^) in their peripheral blood. The accumulation of MDSCs in patients with GBM promotes T-cell immune suppression. Increased plasma levels of arginase and granulocyte colony-stimulating factor may relate to MDSC suppressor function and MDSC expansion, respectively. Removing MDSCs from the PBMCs with anti-CD33/CD15-coated beads significantly restored T-cell function [141].

### 3.5. GBM Stem Cells (GSCs)

Relapse of GBM is attributed to the persistence of hibernating tumor stem cells [142]. The existence of GBM stem cells is also correlated with multidrug and radiation resistance in GBM [143, 144]. In the past few years, one of the neural progenitor cells marker, CD133, was described as a reasonable marker for GSCs as well [145]. However, some GSCs were also reported to be CD133 negative [146]. So the actual concept of GSCs still needs to be defined.

A safe and effective immune response against rodent gliomas can be elicited by using GSC cell lines as a vaccine in rodent models [147]. Glioblastoma-associated antigens such as AIM2, BMI1, COX-2, TRP2, GP100, EGFRv III, EZH2, LICAM, Livin/Livin *β*, MRP3, NESTIN, OLIG2, and SOX2 are present on these human GSCs. In contrast, IL-13R*α*2 and HER2 seem to be decreased in these GSCs [98]. Two studies suggest that GSCs can differentiate into glioma endothelial cells [148, 149]. GSCs might be considered as sources of antigens for DC vaccination against human GBM, with the aim of achieving GSC-targeting and better antitumor immunity [Figure 1].

## 4. Future Perspective

Recently, several studies have reported that the combination of chemotherapy and immunotherapy may be more effective than single-modality treatment alone [150, 151]. Kim et al. demonstrated that in a GL26 glioma model, a combination of low-dose TMZ chemotherapy and transactivation of transcription (TAT)-based DC immunotherapy may be a novel strategy for safe and effective treatment of malignant gliomas. TAT contains a protein transduction domain and could be used as an efficient carrier [152].

Our recent work also showed that TMZ might not be deleterious but rather beneficial to immunomodulatory therapy of GBM patients [153].

Future developments in DC vaccination for GBM might include transfection/nucleofection of DCs with RNA encoding for GAA, cytokines, or TLRs.

## Figures and Tables

**Table 1:** Synopsis of DC-based immunotherapy trials for GBM patients.

Patients	Phase	Route	Antigen format	Immune response	Clinical response	References
22 patients (13 recurrent GBM, 5 AA, 3 AO, 1 AOA)	Phase I/II	Intranodal + intramuscular injections of poly-ICLC	Synthetic peptides for GAAs	Induced positive immune responses against at least one of the GAAs in PBMCs in 58% of patients (after 4 vaccinations). Significant upregulation: interferon-alpha and CXCL10.	4 recurrent GBM are progression free for at least 12 months; 1 CR (recurrent GBM). Median TTP: 4 months.	[61]
23 patients (15 newly diagnosed GBM, 8 recurrent GBM)	Phase I	Intradermal + intramuscular injections of poly-ICLC	Autologous tumor lysate + imiquimod or poly-ICLC	No dose-limiting toxicity. Tumor samples with a mesenchymal gene expression signature had a higher number of CD3^+^ and CD8^+^ tumor-infiltrating lymphocytes	Newly diagnosed: median OS: 35.9 months, with a mean follow-up time of more than 4 years, and 1-, 2-, and 3-year survival rates of 93%, 77%, and 58%, respectively. Recurrent: median OS was 17.9 months from the time of initial glioblastoma diagnosis. OS was significantly longer for those who received DC vaccination at initial diagnosis compared with those who enrolled in this trial at the time of recurrence.	[62]
8 patients (newly diagnosed GBM)	Phase I/II	Intradermal	Autologous tumor lysate	DTH (2/5) increased CD8^+^/CD25^+^ in PBL (6/7) ATR PBMC (5/8) IFN-gamma ELISPOT)	Median OS: 24 months	[63]
45 children (23 recurrent GBM, 5 AA, 1 AOA, 16 other HGG)	Phase I	Intradermal	Autologous tumor lysate + imiquimod	No data available	Median PFS for relapsed GBM: 4.3 months; median OS for relapsed GBM: 12.2 months	[64]
12 patients (newly diagnosed GBM)	Phase I	Intradermal	EGFRvIII antigen + KLH	DTH EGFRvIII (5/9); DTH KLH (9/9); ATR PBMC (10/12) (EGFRvIII-induced proliferation)	Median OS: 22.8 months	[65]
56 patients (recurrent GBM)	Phase I/II	Intradermal	Autologous tumor lysate	DTH (9/21 at time of diagnosis, 9/17 after 2 vaccinations)	3-month PFS; OS: 9.6 months; 24-month OS: 14.8%; total resection is a predictor for better PFS; younger age and total resection are predictors for better OS.	[66]
34 patients (23 recurrent GBM, 11 newly diagnosed GBM)	Phase II	Subcutaneous	Autologous tumor lysate	Postvaccine antigen-directed IFNg response in PBMCs (17/34); DTH-test resulted in cutaneous GBM in 1 patient (DTH was subsequently discontinued)	Newly diagnosed: 8/17 (47%) vaccine responders versus. 3/15 (20%) nonresponders; Recurrent: TTS, 621 ± 81 and 402 ± 49 d; TTP, 28 ± 94 and 142 ± 22 d (8 responders and 13 nonresponders); TTP, 343 ± 116 and 136 ± 19 d (8 responders and 15 nonresponders)	[67]
24 patients (18 recurrent GBM, 6 grade III glioma)	Phase I/II	Intradermal or intradermal + intratumoral (Ommaya reservoir)	Autologous tumor-lysate	DTH to tumor lysate (8/24); ATR PBMC (7/24) (IFN-gamma ELISPOT)	1 PR, 3 MR, 6 SD (GBM); 4 SD (Grade III glioma); median OS: 16 months versus 13.3 months; longer survival if DC maturation or IC injection. One grade IV neurotoxicity event (stupor) observed.	[68]
12 patients (7 newly diagnosed GBM, 5 recurrent GBM)	Phase I	Intradermal	Acid-eluted tumor associated peptides	CTL response (6/12); tumor infiltration CD8^+^ CD45RO^+^ cells (4/8)	Median TTP: 19.9 months—OS 18 to >58 months—median OS: 35.8 months. 1PR; Median OS: 23.4 versus 18.3 months. No dose-limiting toxicity observed	[69]
14 patients (1 newly diagnosed GBM, 9 recurrent GBM, 4 AA)	Phase I	Subcutaneous	Autologous tumor lysate	Increased IFN*γ* RNA in PBMC (6/10) ATR T cells (4/9) (HER-2, gp100, MAGE-1 tetramers); CD8^+^, CD45RO^+^ cells infiltration (3/6)	Median survival: 33.3 versus 7.5 months (8/9 recurrent GBM).	[70]
15 patients (6 recurrent GBM, 7 AA, 2 OAA)	Phase I	Intradermal	DC fusion with autologous glioma cells	DTH to tumor lysate (15/15); increased cytotoxic activity (2/15); increased intracellular IFN-gamma in CD8^+^ T cells (1/15)	1 SD (GBM); 3 PR, 1 MR (AA); 1 PR, 1 SD (AOA)	[71]
7 patients (2 recurrent GBM, 1 AA, 4 other HGG)	Phase I	Intradermal	Autologous tumor RNA	No anti-tumor responses (0/3) (IFN-gamma ELISA)	1 PR (1XA); 4 SD (1AA, 3 other HGG)	[72]
25 patients (newly diagnosed GBM: 13 plus chemotherapy, 12 without chemotherapy)	Phase I/II	Intradermal	Autologous tumor lysates or peptide elutions	Vaccine alone: ATR PBMC (4/11) Vaccine + chemotherapy: ATR PBMC (4/13) (lytic activity and IFN-gamma PCR)	Vaccine or chemotherapy alone: 24-month survival: 8% Vaccine + chemotherapy: 3 PR; 24-month survival: 42%	[73]
10 patients (7 recurrent GBM after radiotherapy, 3 recurrent grade III glioma)	Phase I/II	Intradermal and/or intratumor (Ommaya)	Autologous tumor lysate	Increase in NK cells in PBMCs (5/10); DTH to tumor lysate (3/10); increased T-cell mediated antitumor activity (2/10)	2 MR, 2SD (GBM), 2SD (Grade III glioma); OS > 50 months.	[74]
9 patients (7 newly diagnosed GBM, 2 AA after radiotherapy)	Phase I	Subcutaneous	Tumor-specific MHC-I-associated peptides	Systemic CTL cytotoxicity against tumor (4/9) (lytic activity); tumor infiltration: CD4^+^, CD8^+^, CD45RO^+^ cells (2/4)	Prolonged median survival compared to control group: 15.2 versus 8.6 months (GBM)	[75]

Abbreviations: AA: anaplastic astrocytoma; AO: anaplastic oligodendroglioma; AOA: anaplastic oligoastrocytoma; ATR: anti-tumor responses; CR: complete response; DTH: delayed-type hypersensitivity; GAA: glioblastoma associated antigen; GBM: glioblastoma multiform; HGG: high-grade glioma; KLH: keyhole limpet haemocyanin; MR: Minor response; OS: overall survival; PBMC: peripheral blood mononuclear cells; PFS: progression-free status; PR: partial response; PXA: pleomorphic xanthoastrocytoma; SD: stable disease; TMZ: Temozolomide; TTP: Time to tumor progression; TTS: Time to tumor survival; XA: xanthoastrocytoma.

**Table 2:** List of glioblastoma-associated antigens (GAAs).

GAAs	Characteristic/potential function	References
*AIM2: absent in melanoma 2	AIM-2 could be used as a tumor antigen target for monitoring vaccine trials or for developing antigen-specific active immunotherapy for glioma patients.	[90, 96–98]
*BMI1: BMI1 polycomb ring finger oncogene	Expressed in human GBM tumors and highly enriched in CD133^+^ GSC cells.	[99]
*COX-2: cyclooxygenase-2	Overexpressed in many tumors including CD133^+^ GSC cells, COX-2 inhibitor celecoxib will become a nice weapon for GBM therapy.	[100]
*TRP-2: tyrosinase related protein 2	Highly expressed in GSCs.	[89, 97, 98]
*GP100: glycoprotein 100	Melanocyte lineage-specific antigen, expressed in GSCs as well.	[89, 98]
*EGFRvIII: epidermal growth factor receptor variant III	EGFRvIII is the most prevalent of several EGFR mutations found in human gliomas and is expressed in 20–25% of GBM. GSC-associated antigen.	[79, 89, 98]
*EZH2: enhancer of zeste homolog 2	Upregulated in malignant gliomas and in GSC cells.	[97, 101]
*LICAM: human L1 cell adhesion molecule	Highly expressed in GSCs. Invasion-associated proteins.	[102]
*Livin and Livin*β*	The expression of livin and livin*β* in CD133^+^ U251 stem-like cells was much higher than that in cancer cells, Livin*β* was more related with the high survival rate. It is a cancer-associated member of the inhibitor of apoptosis protein (IAP).	[103]
*MRP-3: multidrug-resistance protein 3,	GBMs overexpress MRP3 at both mRNA and protein levels. Multidrug-resistance protein 3, potential correlation with survival. Highly expressed in GSC cells as well.	[97, 98, 104]
*Nestin	Nestin plays important roles in cell growth, migration, invasion, and adhesion to extracellular matrices in glioma cells. Overexpressed in GSCs.	[105]
*OLIG2: oligodendrocyte transcription factor 2	GSC marker, OLIG2 is highly expressed in all diffuse gliomas. Immunohistochemistry and microarray analyses demonstrated higher OLIG2 in anaplastic oligodendrogliomas versus glioblastomas, which are heterogeneous with respect to OLIG2 levels.	[106]
*SOX2: SRY-related HMG-box 2	SOX2 expression and amplification in gliomas and GSC cell lines.	[98, 107]
ART1: antigen recognized by T cells 1	Pediatric GBM express ART1, ART4, SART1, SART2, and SART3, they were identified within glioblastoma cell lines as well.	[92, 97]
ART4: antigen recognized by T cells 4		
SART1: squamous cell carcinoma antigen recognized by T cells 1		
SART2: squamous cell carcinoma antigen recognized by T cells 2		
SART3: squamous cell carcinoma antigen recognized by T cells 3		
B-cyclin	Overexpressed in GBM.	[97, 108]
*β*-catenin	*β*-catenin and Gli1 are prognostic markers in GBM.	[109]
Gli1: glioma-associated oncogene homolog 1	Gli1 is correlated with glioma recurrence after chemotherapy, Gli1 plays a dominant role in chemoresistance of glioma cells. located in nuclear, might be fluctuating between the cytoplasm and the nucleus.	[109, 110]
Cav-1: caveolin-1	Expressed in most HGG, correlated with proliferation and invasive potential of tumor.	[111]
Cathepsin B	Overexpression of cathepsin B during the progression of human gliomas.	[112]
CD74: cluster of Differentiation 74	Contribute to TMZ resistance. Also known as HLA class II histocompatibility antigen gamma chain.	[113]
E-cadherin: epithelial calcium-dependent adhesion	Expression in gliomas correlated with an unfavorable clinic outcome.	[114]
EphA2/Eck: EPH receptor A2/epithelial cell kinase	Overexpressed in both pediatric and adult GBM. Used as a novel target for glioma vaccines.	[90, 97, 115]
Fra-1/Fosl 1: fos-related antigen 1	Plays an important role in maintenance/progression of various cancers, including GBM. Highly expressed in pediatric GBM.	[97, 116]
GAGE-1: G antigen 1	A potential target for specific immunotherapy and diagnostic markers in high-grade brain tumors.	[93]
Ganglioside/GD2	Expressed in astrocytic tumors.	[117]
GnT-V, *β*1,6-N: acetylglucosaminyltransferase-V	Plays an important role in regulating invasivity of human glioma.	[97, 118]
Her2/neu: human epidermal growth factor receptor 2	A tumor-associated antigen that is expressed by up to 80% of GBMs but not by normal postnatal neurons or glia.	[97, 98]
Ki67: nuclear proliferation-associatedantigen of antibody Ki67	Prognostic marker for glioma, especially for the lower grades.	[79]
Ku70/80: human Ku heterodimer proteins subunits (molecular weight: 70 kDa/80 kDa)	A therapeutic potential target antigen. Highly expressed in GBM.	[119]
IL-13R*α*2: interleukin-13 receptor subunit alpha-2	Overexpressed in GBM but diminished in several GSC cell lines.	[89, 97, 98]
MAGE-A: melanoma-associated antigen 1	MAGE-A1, MAGE-A3, and NY-ESO-1 can be upregulated in neuroblastoma cells to facilitate cytotoxic T-lymphocyte-mediated tumor cell killing.	
MAGE-A3: melanoma-associated antigen 3		[94]
NY-ESO-1: New York oesophageal squamous cell carcinoma 1		
MART-1: melanoma antigen recognized by T-cells	Melanoma antigen also associated with glioma.	[95]
PROX1: prospero homeobox protein 1	Strongly express in GBM, frequently coexpress early neuronal proteins MAP2 and betaIII-tubulin but not the mature neuronal marker NeuN.	[120]
PSCA: prostate stem cell antigen	GPI-anchored cell surface protein, represented as a novel GAA.	[121]
SOX10: SRY-related HMG-box 10	The SOX10 expression was restricted to gliomas and melanomas. All glioma types expressed SOX10, and tumors of low-grade glioma had a much broader distribution of SOX10 compared with high-grade gliomas.	[122]
SOX11: SRY-related HMG-box 11	The transcription factor SOX11 highly specific overexpression of in human malignant gliomas.	[87, 97]
Survivin	Quantitatively determined survivin expression levels are of prognostic value in human gliomas.	[79, 97]
UPAR: urokinase-type plasminogen activator receptor	UPAR and Cathepsin B, known to be overexpressed in high-grade gliomas and strongly correlated with invasive cancer phenotypes.	[123]
WT-1: Wilms' tumor protein 1	A transcription factor overexpressed in glioma.	[91]

*: Glioblastoma stem cell (GSC) associated antigens as potential targets for immunotherapy.
